# Neuroprotective Effects of Isosteviol Sodium Injection on Acute Focal Cerebral Ischemia in Rats

**DOI:** 10.1155/2016/1379162

**Published:** 2016-03-07

**Authors:** Hui Hu, Xiao ou Sun, Fang Tian, Hao Zhang, Qing Liu, Wen Tan

**Affiliations:** ^1^Pre-Incubator for Innovative Drug & Medicine, South China University of Technology, Guangzhou 510006, China; ^2^Guangdong Provincial Key Laboratory of Fermentation and Enzyme Engineering, School of Bioscience & Bioengineering, South China University of Technology, Guangzhou 510006, China

## Abstract

Previous report has indicated that isosteviol has neuroprotective effects. However, isosteviol was administered preventively before ischemia and the inclusion criteria were limited. In the present study, a more soluble and injectable form of isosteviol sodium (STVNA) was administered intravenously hours after transient or permanent middle cerebral artery occlusion (tMCAO or pMCAO) to investigate its neuroprotective effects in rats. The rats were assessed for neurobehavioral deficits 24 hours after ischemia and sacrificed for infarct volume quantification and histology evaluation. STVNA 10 mg·kg^−1^ can significantly reduce the infarct volumes compared with vehicle in animals subjected to tMCAO and is twice as potent as previously reported. Additionally, the therapeutic window study showed that STVNA could reduce the infarct volume compared with the vehicle group when administered 4 hours after reperfusion. A similar effect was also observed in animals treated 4 hours after pMCAO. Assessment of neurobehavioral deficits after 24 hours showed that STVNA treatment significantly reduced neurobehavioral impairments. The number of restored NeuN-labeled neurons was increased and the number of TUNEL positive cells was reduced in animals that received STVNA treatment compared with vehicle group. All of these findings suggest that STVNA might provide therapeutic benefits against cerebral ischemia-induced injury.

## 1. Introduction

Stroke is the second leading cause of death worldwide in people aged over 60 years [1]. Ischemic stroke accounts for 70% of all strokes [2]. Ischemic stroke is the result of a transient or permanent fall in cerebral blood flow, which is restricted to the territory of a major brain artery [3]. Though timely recanalization of the occluded vessel is an effective treatment, it can result in brain injuries, such as cerebral edema, parenchymal hemorrhage, and neuronal death. Moreover, there are no FDA approved neuroprotectants to treat ischemic stroke.

Isosteviol sodium, the sodium salt of isosteviol, is a beyerane diterpene obtained by the acid hydrolysis of stevioside [4, 5]. Stevioside, a major component of the* Stevia rebaudiana* leaf, is used as a conventional noncaloric sweetener and has been used in traditional medicine for several hundred years [6, 7]. Several studies indicate that isosteviol possesses a variety of biological activities including antihypertension [8], antihyperglycemia [9], antioxidant [10], anti-inflammatory [11], and antitumor effects [12] and relieves ischemia-reperfusion injury in the rat brain [13, 14].

In previous studies, isosteviol was administered before ischemia and these studies were not designed according to the suggestions of the Stroke Therapy Academic Industry Roundtable [15]. Isosteviol is also only slightly soluble in water and this low solubility affects its bioavailability. Thus, it is difficult to use isosteviol as an aqueous injection, which limits its applications in emergency treatment. STVNA is an injectable formulation of the isosteviol sodium salt dissolved in a mixture containing water and organic solvents and has increased bioavailability compared with isosteviol alone. Therefore, the present study was designed to demonstrate the possible therapeutic effects of STVNA in focal acute ischemia/reperfusion (IR) injury in rats.

## 2. Materials and Methods

### 2.1. Animals

Adult male Sprague-Dawley rats weighing 250–280 g were purchased from the Experimental Animal Center of Sun Yat-sen University (Guangzhou, China). The rats were housed four per cage in a room under controlled temperature, humidity, and 12-hour light/12-hour dark cycles with free access to food and water. The rats were allowed to acclimatize for at least three days before the experiment. All of the experimental procedures complied with current national and international laws and recommendations, and the study was approved by the Institutional Animal Care and Use Committee of Sun Yat-sen University.

### 2.2. Middle Cerebral Artery Occlusion Model

The middle cerebral artery occlusion (MCAO) was performed through an intraluminal suture as previously described [16]. Occlusion of the right MCA was induced either transiently for 2 hours (tMCAO) followed by a reperfusion period of 22 hours or permanently for 24 hours (pMCAO). Briefly, anesthesia was induced in rats with 3% isoflurane and was maintained with 2% isoflurane in a gas mixture of 5% CO_2_ and 95% O_2_. The temperature was maintained constant at (37.0 ± 0.5)°C by a thermostatically controlled heating blanket throughout the surgical procedure. The right common carotid artery and the internal carotid artery were exposed under an operative microscope through a neck midline incision. The pterygopalatine artery was ligated close to its origin. A 3-0 nylon filament suture coated at the tip with 5 mm of silicone was inserted into the common carotid artery and up to the internal carotid artery for a distance of 19 to 21 mm from the common carotid artery bifurcation. After 2 hours of ischemia, the nylon suture was removed to allow reperfusion period for 22 hours in the case of the tMCAO or the occlusion was maintained permanently for 24 hours in the case of the pMCAO. The incision was closed, and the rats were returned to the cages after they awakened from anesthesia. The middle cerebral artery blood flow was monitored in real time by laser-Doppler flowmetry (PeriFlux System 5000, Perimed AB, Stockholm, Sweden) 2.5 mm lateral and 2.0 mm posterior to the bregma during preischemia, postischemia, and reperfusion periods to verify the success of the cerebral ischemia/reperfusion procedure. Physiological parameters including heart rate, breathing rate, and SpO_2_ (Pulse Oximeter Oxygen Saturation) were monitored during the surgical procedure by a MouseOx Plus Pulse Oximeter (STARR Life Sciences Corp., Oakmont, USA). Sham animals were subjected to the same surgical procedures except that the suture was not advanced into the middle cerebral artery.

### 2.3. Drugs and Treatment Schedule

STVNA was provided by the Chemical Development Laboratories of Key Biological Pharmaceutical Company (Dongguan, China). Edaravone was purchased from Simcere Pharmaceutical Co., Ltd. (Nanjing, China).

In all of the experiments, the drugs were administered by an i.v. drip at a rate of 0.25 mL/min for 1 min (loading infusion) followed by a rate of 0.25 mL/h for 3 hours (maintaining infusion) with a micro-perfusion pump (LSP04-1A, Longer Precision Pump Co., Ltd., Baoding, China). First, the femoral vein was carefully exposed and two silk ligatures were placed around the vein by blunt dissection. A small incision was then made in the femoral vein and a cannula (PE-10) was inserted to a depth of 3.0 cm. The cannula was then securely held in place by tying two ligatures around the cannulated femoral vein. A hard tube was used to sheathe the vein cannula to prevent the animal from biting it. The animal was limited into a Plexiglas box for 3 hours, and when the infusion was finished, they were placed back in the cages.

The STVNA dose-response relationship was investigated in the transient ischemia model (tMCAO). STVNA (1, 5, 10, and 20 mg/kg, *N* = 11 per group), edaravone (3 mg/kg, *N* = 11), and vehicle (*N* = 11) were administered to the respective groups of rats subjected to tMCAO starting 1 hour before reperfusion. Solvent was administered to sham animals 1 hour before reperfusion (*N* = 11). The STVNA therapeutic window was investigated at a single dose of 10 mg/kg in both transient (tMCAO) and permanent (pMCAO) ischemia models. In the tMCAO experiments, STVNA was administered at 0, 2, 4, or 6 hours after reperfusion (*N* = 8 per group). Vehicle was delivered 2 hours after MCAO (*N* = 8). In the pMCAO experiments, STVNA was administered at 0, 2, 4, or 6 hours after the middle cerebral artery occlusion (*N* = 8 per group). Vehicle was delivered 2 hours after MCAO (*N* = 8). Moreover, in the measurement of brain edema experiments, STVNA (10 mg/kg) was administered 1 hour before reperfusion (*N* = 6). Sham and vehicle were delivered 1 hour after MCAO (*N* = 6 per group). Rats were randomly assigned to each experimental group before MCAO.

### 2.4. Evaluation of Neurological Deficits

Twenty-four hours after MCAO, rats were tested for neurological deficits according to Bederson et al.'s test [17]. The scores were assigned using the following scale: 0, no neurological deficits; 1, failure to fully extend the right forepaw; 2, circling to the right; 3, falling to the right; and 4, absence of spontaneous walking and depressed levels of consciousness. The investigator applying the behavior test did not know the identity of the experimental treatment groups.

### 2.5. Measurement of Cerebral Infarction

Rats were euthanized by intraperitoneal injection of 100 mg/kg sodium pentobarbital 24 hours after MCAO. Then, the brain tissue was removed and sliced into 2.0 mm thick sections using a brain matrix (J&K Seiko Electronic, Dongguan, China). The brain slices were incubated for 10 min at 37°C in 2% triphenyltetrazolium chloride (TTC, Sigma-Aldrich, Saint Louis, USA) dissolved in saline solution. The TTC-stained coronal sections from each individual were scanned with a color flatbed scanner (Scanjet G3100, Hewlett-Packard, Shanghai, China). Infarction volume was measured using the ImageJ v1.48 software (US National Institutes of Health, Bethesda, Maryland, USA, http://imagej.nih.gov/ij/) and calculated as previously described [18].

### 2.6. Measurement of Edema

Twenty-four hours after stroke, cerebral edema was determined by comparing the wet-to-dry tissue weight ratios as previously described [19]. Briefly, the brain was quickly removed after the animal was sacrificed. Then, the brain was blotted to remove residual moisture and dissected through the interhemispheric fissure into right and left hemispheres. Samples were immediately weighed to obtain the wet weight. The dry weight was obtained after drying for 2 days at 120°C. The water content of both hemispheres was determined using the following equation: brain water content (%) = (wet weight − dry weight) × 100/wet weight.

### 2.7. Tissue Preparation and HE Staining

To evaluate the histological damage, three animals in each group from the dose-response study were sacrificed after 22 hours of reperfusion and were perfused with 100 mL of saline solution, followed by 100 mL of freshly prepared 4% (v/v) paraformaldehyde in 0.01 M phosphate buffered saline (PBS, pH 7.4). The brain was then removed and fixed in 10% (w/v) paraformaldehyde in 0.01 M PBS for 1 week. Then, a 4 mm coronal section of the brain was cut 2.0 mm anterior and posterior to the bregma and the block was embedded in paraffin. The block was then cut into 5 *μ*m coronal sections that were stained with hematoxylin-eosin (HE) using standard methods.

### 2.8. Immunohistochemistry Assays

Coronal brain sections (5 *μ*m thick) were randomly selected between 0 and 2.0 mm posterior to the bregma. After deparaffinization and rehydration, the sections were incubated for 10 min at 95–98°C in 0.01 M citrate buffer (pH 6.0) for antigen retrieval. The sections were allowed to cool down to room temperature (RT) and were then incubated for 15 min in H_2_O_2_ (1% in 0.01 M PBS, v/v) and 0.5 hours in 5% bovine serum albumin (Sigma, St. Louis, USA) blocking solution (5% in 0.01 M PBS, w/v) at RT. Then, the sections were incubated overnight at 4°C in a solution of anti-NeuN antibody (1 : 500, v/v, clone A60, MAB377, Merck Millipore, Darmstadt, Germany). The sections were washed with 0.01 M PBS and then incubated with the Mouse EnVision+ System-HRP (K4006, DAKO, Glostrup, Denmark) for 1 hour at RT. Positive staining was visualized with 3,3′-diaminobenzidine (DAB) using a DAB-enhanced liquid substrate system (DAKO, Glostrup, Denmark). Finally, the sections were counterstained with hematoxylin, dehydrated, mounted, and observed under a microscope (Leica DM4000 BLED, Wetzlar, Germany). The number of immunopositive cells was counted in five randomly selected fields from the peri-infarct area per slice and presented as the number of cells/mm^2^. All of the positively stained cells (brown color) were included in the count regardless of their morphology.

### 2.9. TUNEL Assays

Coronal sections between 0 and 2.0 mm posterior to the bregma were chosen. For the detection of neuronal cell death, in situ nick end labeling was performed using a commercially available kit (ApopTag^®^ Plus Peroxidase In Situ Apoptosis Detection Kit S7101, Millipore, Darmstadt, Germany) according to product's specifications. Briefly, after dewaxing in water, tissue sections were washed in PBS (0.01 M, pH 7.4) for 5 min, permeabilized by using proteinase K (20 *μ*g/mL in 0.01 M PBS) for 10 min, and quenched for 5 min in H_2_O_2_ (3% in 0.01 M PBS, v/v) at RT. Then, the sections were incubated in equilibration buffer for 20 min and labeled overnight at 4°C; the reaction was stopped by the addition of stop buffer. Sections were rinsed with PBS (0.01 M, pH 7.4) and incubated in peroxidase streptavidin conjugate for 45 min. TUNEL positive cells were visualized with a DAB kit. Finally, the sections were counterstained with hematoxylin, dehydrated, and mounted. TUNEL positive cells were observed at an effective magnification of 400x in the measured areas and photographed using a digital camera (Leica DM4000 BLED, Wetzlar, Germany). The number of TUNEL positive cells throughout the ipsilateral hemisphere was counted in five fields from random peri-infarct areas per slice and presented as the number of cells/mm^2^. All of the positively stained cells (brown color) were included in the count regardless of their morphology.

### 2.10. Statistical Analyses

Data were presented as the mean ± the SEM. Differences between groups were examined using the one-way analysis of variance (ANOVA) with Tukey's multiple comparison test. A* p* value less than 0.05 was often reported as statistically significant.

## 3. Results

### 3.1. Cerebral Blood Flow

**Figure 1:**
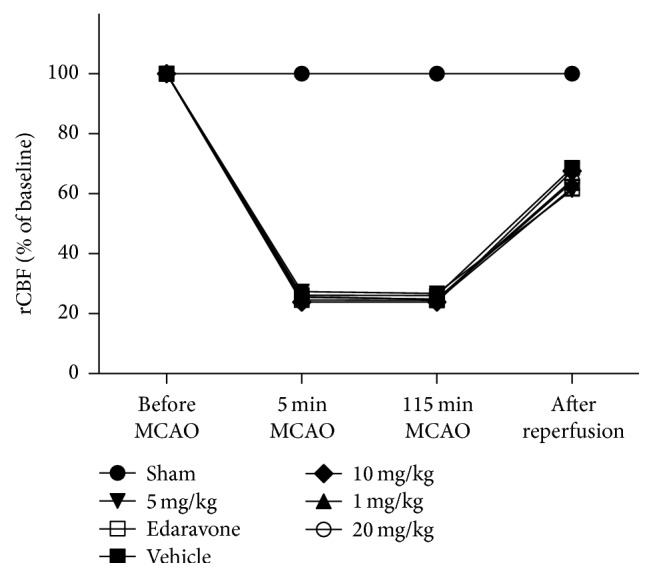
Regional cerebral blood flow (rCBF) before and during middle cerebral artery occlusion (MCAO) and after reperfusion. The rCBF was monitored 5 min before the transient middle cerebral artery occlusion (MCAO) and at 5 and 115 min during MCAO and 5 min after reperfusion. Monitoring of rCBF insured that the MCAO model was successful. Sham = sham group; vehicle = model group. The 1 mg/kg, 5 mg/kg, 10 mg/kg, and 20 mg/kg represent different treatment dosage groups. All of the data are shown as the mean ± the SEM (*n* = 8).

The regional cerebral blood flow (rCBF) value during ischemia decreased to less than 30% of the baseline value in all of the groups except for the sham group in the dose-response study (Figure 1) and remained constant during the ischemic period in rats. The rCBF of the vehicle group was not significantly different compared with the STVNA-treated groups at the same time points.

### 3.2. Physiologic Variables

All of the animals in this study showed similar values for heart rate, breathing rate, and SpO_2_ (Table 1). Although tMCAO caused transient and mild hyperthermia approximately 120 minutes after the occlusion, that effect was not changed by STVNA.

### 3.3. STVNA Alleviated Brain Edema in the Focal Ischemia Rats

**Figure 2:**
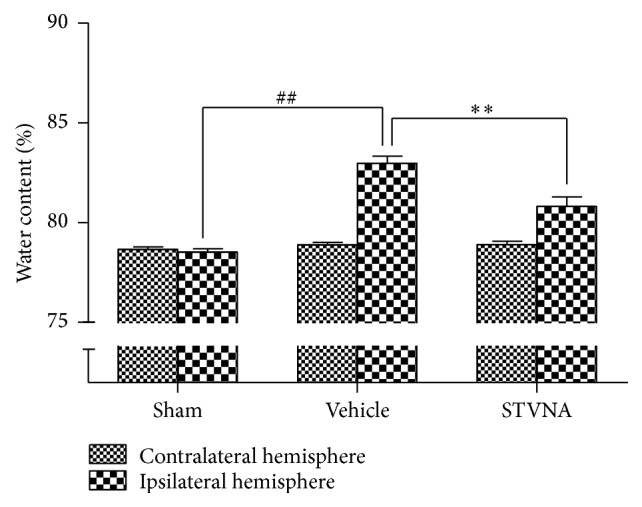
Hemispheric water content. Water content in the ischemic and nonischemic contralateral brain hemispheres studied 24 hours after 2 hours of MCAO in rats with and without STVNA administration. STVNA significantly inhibited edema formation in the ischemic hemisphere. Histograms represent the mean ± the SEM (*n* = 6). ^##^ *p* < 0.01 versus sham group; ^*∗∗*^ *p* < 0.01 versus vehicle group by 1-way analysis of variance with Tukey's multiple comparison test.

As shown in Figure 2, 2 hours of MCAO and 22 hours of reperfusion resulted in an evident increase in brain water content in the ipsilateral hemisphere compared with the sham-operated rat brain (*p* < 0.01). The brain water in the ipsilateral hemisphere observed in the vehicle group (82.9 ± 0.9%) was significantly reduced to 80.8 ± 1.2% in the STVNA-treated group, while the water content in the contralateral hemisphere did not show significant differences between the two groups. Briefly, STVNA treatment (10 mg/kg) significantly reduced the brain water content compared with the vehicle group.

### 3.4. Dose-Response Study

**Figure 3:**
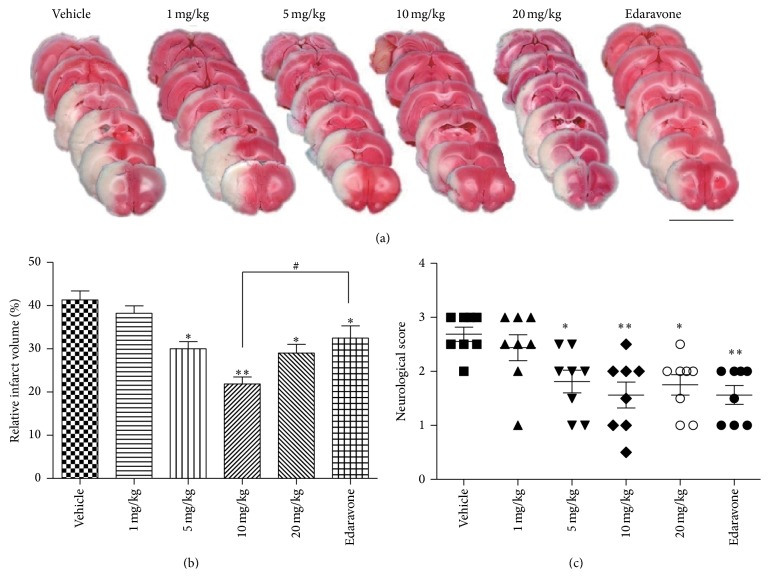
Effects of STVNA on various parameters measured 24 hours after tMCAO in rats. (a) Infarction in serial brain sections stained by TTC (magenta: healthy tissue; white: damaged tissue). Scale bar = 0.5 cm. (b) Statistical analysis of the percentage of infarct volume was determined for each group. (c) Neurological scores after transient middle cerebral artery occlusion (tMCAO) in the vehicle and STVNA treatment groups and edaravone group. Data were expressed as the mean ± the SEM (*n* = 8 per group). ^∗^ *p* < 0.05 and ^*∗∗*^ *p* < 0.01 versus vehicle group; ^#^ *p* < 0.05 versus edaravone group by 1-way analysis of variance with Tukey's multiple comparison test.

Figure 3 shows the dose-response features of the histological and functional amelioration induced by STVNA. Infarct volume was calculated after 24 hours using TTC staining (Figure 3(a)). A significant reduction in infarct size was already observed at 5 mg/kg (30.6 ± 1.6%), and the highest reduction was obtained at 10 mg/kg (22.7 ± 1.5%) compared with the vehicle group (41.3 ± 2.1%), whereas the 1 mg/kg (38.2 ± 1.7%) dose was ineffective and the 20 mg/kg (29.1 ± 1.9%) dose did not contribute to further alleviation of the infarct volume (Figure 3(b)). Edaravone, a reactive oxygen species scavenger approved as a neuroprotectant in Japan for the treatment of cerebral infarction, was used as a positive control. The results indicate that 10 mg/kg STVNA was significantly more effective than edaravone (22.7 ± 1.5% versus 32.4 ± 2.8%, *p* < 0.05) in reducing the infarct size.

To determine whether the histological improvement offered by STVNA had effects on ischemic behavioral patterns, we evaluated neurological deficits 24 hours after transient ischemia. The scores obtained in the groups treated with 5 mg/kg and 10 mg/kg of STVNA were significantly better than those in the animals treated with vehicle (1.8 ± 0.2 and 1.5 ± 0.2 versus 2.6 ± 0.1, resp.) (Figure 3(c)).

### 3.5. Therapeutic Window Study

**Figure 4:**
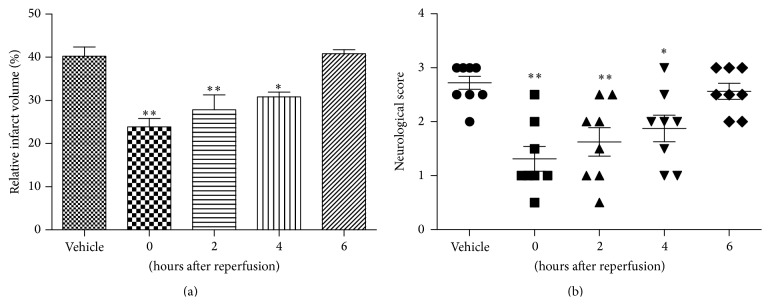
Therapeutic window characteristics of STVNA treatment in tMCAO. (a) Quantitative analyses of infarct volumes 24 hours after reperfusion. A high infarction volume was seen in the group without STVNA treatment, but the volume was greatly decreased in rats with STVNA administration in a time-dependent manner. (b) Neurologic scores after transient middle cerebral artery occlusion (MCAO) in different groups with STVNA administered at 0, 2, 4, and 6 hours after reperfusion. Neurological deficit scores 24 hours after reperfusion. A high score was seen in the group without STVNA treatment, but the scores were greatly lowered in animals with STVNA administration. Data represent mean ± SEM (*n* = 8 per group). ^∗^ *p* < 0.05; ^∗∗^ *p* < 0.01 versus vehicle group by 1-way analysis of variance with Tukey's multiple comparison test.

A systematic study was performed to examine the time interval after the induction of transient ischemia at which STVNA was still capable of protecting the brain. The vehicle was immediately administered after reperfusion and 10 mg/kg STVNA was administered at 0, 2, 4, or 6 hours after reperfusion. A significant reduction of the infarct volumes was observed when STVNA was administered 2 hours (27 ± 3.5%) or 4 hours (31 ± 1.2%) after reperfusion compared with the vehicle group (41.3 ± 2.1%), whereas no significant effect was observed when the administration time was delayed to 6 hours (40.8 ± 0.9%) (Figure 4(a)). Consistently, a remarkable improvement in neurological scores was detected in the STVNA-treated group (Figure 4(b)).

### 3.6. Permanent Ischemia Study

**Figure 5:**
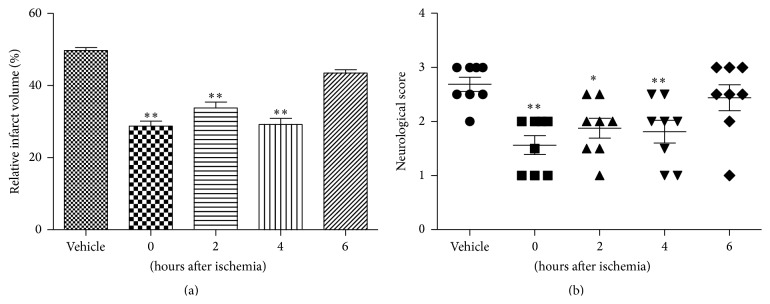
Effects of STVNA treatment in pMCAO. (a) The infarct volume of each group 24 hours after pMCAO. The volume was greatly decreased in rats treated with STVNA administered 4 hours after ischemia. (b) Neurological scores after permanent middle cerebral artery occlusion (pMCAO) in different groups with STVNA administered at 0, 2, 4, and 6 hours after ischemia. The neurological scores were greatly lowered in animals to which STVNA was administered 4 h after ischemia. The data represent the mean ± the SEM (*n* = 8 per group). ^∗^ *p* < 0.05, ^∗∗^ *p* < 0.01 versus vehicle group by 1-way analysis of variance with Tukey's multiple comparison test.

In permanent MCAO study, STVNA (10 mg/kg) was administered to rats 0, 2, 4, or 6 hours after ischemia induction. In the permanent MCAO model, the tissue damage observed in the contralateral hemisphere was greater than that seen in the transient MCAO (50 ± 0.8% versus 41.3 ± 2.1%) 24 hours after vehicle treatment. Treatment with STVNA showed a statistically significant reduction of ischemic injury when applied up to 4 hours after MCAO (Figure 5(a)). In particular, the treatment with STVNA 4 hours after the occlusion still significantly reduced the infarct volume compared with the vehicle group (29 ± 1.7% versus 50 ± 0.8%, *p* < 0.05); likewise the administration after 6 hours resulted in decreased damage, although this difference was not statistically significant. The neurological deficit was significantly improved in rats treated with STVNA compared with those treated with the vehicle (Figure 5(b)).

### 3.7. Histopathology Analyses

**Figure 6:**
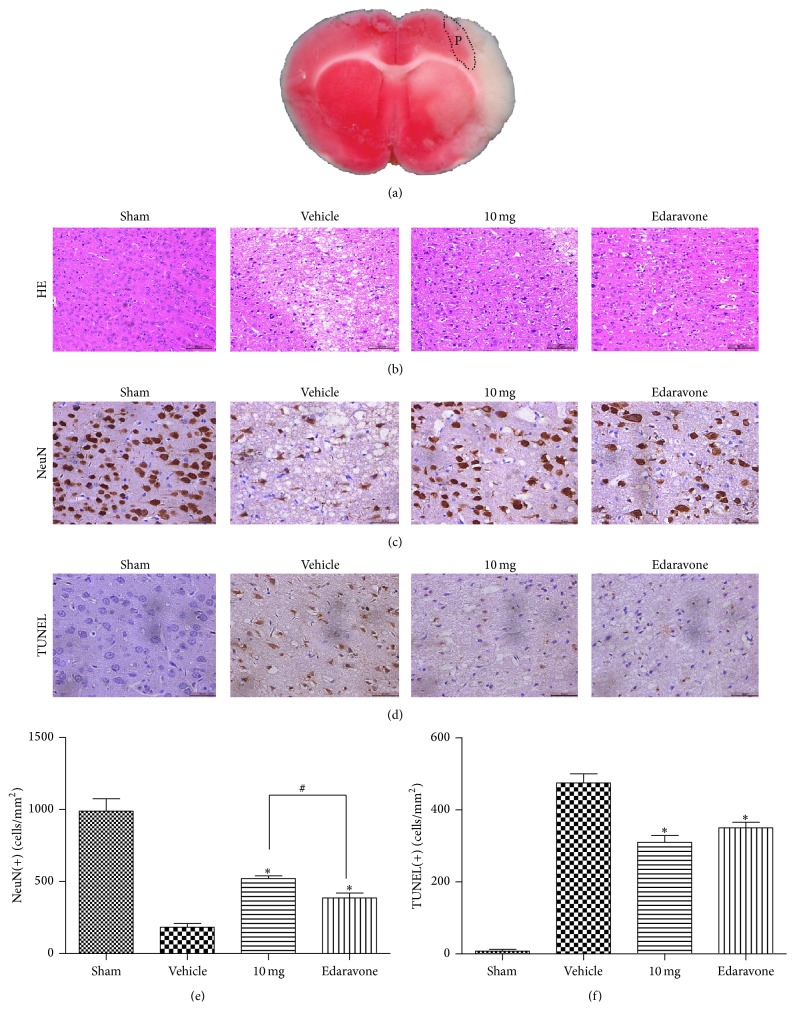
Effects of STVNA on the histopathology. (a) Brain sections stained by TTC; the P represents the peri-infarct area. (b) Hematoxylin-eosin stains of coronal sections of the brain after 24 hours of reperfusion in the dose-response study groups (*n* = 3 per group). 10 mg: 10 mg/kg STVNA. Scale bar = 100 *μ*m. (c) Effects of STVNA on neuronal immunoreactivity in tMCAO rats (*n* = 3 per group). Scale bar = 50 *μ*m. (d) Effect of STVNA on apoptosis in the rat ischemic brain (*n* = 3 per group). Scale bar = 50 *μ*m. (e) Number of NeuN-immunopositive cells/mm^2^ of brain section. Histograms represent the mean ± the SEM. ^∗^ *p* < 0.05 versus vehicle group and ^#^ *p* < 0.05 versus edaravone group by 1-way analysis of variance with Tukey's multiple comparison test. (f) Number of TUNEL positive cells/mm^2^ in the brain sections. Histograms represent the mean ± the SEM. ^∗^ *p* < 0.05 versus vehicle group by 1-way analysis of variance with Tukey's multiple comparison test.

The results from the HE staining are shown in Figure 6(b). In the sham group, the brain tissues were undamaged, cortical neurons were normomorphic, and the nuclei were centered and displayed clear staining. In the model group, most of the neurons from the peri-infarct area appeared shrunken with eosinophilic cytoplasm accompanied by a decreased optical density. However, when treated with STVNA (10 mg/kg) and edaravone, the number of normal neurons significantly increased and the extent of the damage was significantly diminished.

The number of neurons in the peri-infarction area was detected by anti-NeuN immunohistochemistry (Figure 6(c)). The ischemic injury model (tMCAO) showed a clear decrease in NeuN staining compared with the sham group. Treatment with STVNA showed a clear increase in NeuN immunoreactivity and a significant decrease in the extent of damage, even when compared with edaravone (Figure 6(e)).

Furthermore, we performed TUNEL assays to verify whether STVNA treatment reduced apoptosis. The brain sections obtained from the sham group showed only a slight background staining (Figure 6(d)) with no TUNEL positive cells. The brain sections obtained from the vehicle groups showed an increase in the number of TUNEL positive cells, which appeared dark brown. The increase in TUNEL positive cells was inhibited by STVNA treatment. In particular, the reduction of TUNEL positive cells was found to be 34.9% and 26.2% in the STVNA- (10 mg/kg) and edaravone-treated groups, respectively (Figure 6(f)).

## 4. Discussion

In the current study, we demonstrated the dose-response relationship and defined the therapeutic window for the neuroprotective effects of STVNA in tMCAO and pMCAO. The presented data show that moderate doses of STVNA (5, 10, and 20 mg/kg) have a protective effect against neuronal damage induced by cerebral ischemia and that this protection is associated with an improvement in neurological functions. Furthermore, the data showed that STVNA was still effective even if the treatment was delayed up to 4 hours after recirculation in temporary focal ischemia or up to 4 hours after artery occlusion in permanent ischemia.

In the first part of this investigation, the results showed that STVNA posttreatment at 10 mg/kg dramatically reduced the infarct size and neurological deficits. These data are consistent with a previously published study that demonstrated the neuroprotective efficacy of isosteviol after ischemia/reperfusion injury when administered before ischemia at doses of 5 to 20 mg/kg. The rCBF of the animals was not monitored, which was very important for producing a stable model in the previous study. The rCBF was monitored in our study. Furthermore the drug we used is more water soluble than isosteviol; thus, 10 mg/kg STVNA in our study was as effective as 20 mg/kg of isosteviol in Xu et al.'s study [14]. Additionally, we changed the method of STVNA administration to continuous intravenous injection instead of bolus, and the time of drug administration was delayed up to 1 hour after ischemia, which caters to the requirements of clinical therapy. Moreover, the best dosage of the STVNA was reduced by half compared with isosteviol (20 mg/kg) with more security, and the efficacy of STVNA is better than that of isosteviol in reducing the infarct volume percentage (45% versus 33%).

Another significant observation made in our study was the beneficial therapeutic window of STVNA using a clinically relevant route in a transient ischemia model. Generally, it is difficult to get patients to the hospital, evaluate them, and enroll them in a clinical trial within 3 hours of symptom onset. Thus, whether a candidate drug has an advantageous therapeutic window is crucially important in preclinical studies, and rodent studies appear to be relevant for determining a therapeutic window for neuroprotective drugs [20, 21]. Here we found that the favorable effects of STVNA were still present even when the compound was administered 4 hours after the recirculation in transient ischemia or 4 hours after artery occlusion in permanent ischemia. This indicates that STVNA is effective in rodents in a time window that is relevant to clinical practice. We also found that when STVNA was administered at the onset of reperfusion, the infarct volume was not significantly different compared to when STVNA was administered 1 hour before reperfusion (23.8 ± 1.9% versus 22.7 ± 1.5%, *p* > 0.05). As we know that reperfusion may be more harmful to tissues than ischemia [22, 23], our results suggest that STVNA may be more effective in preventing reperfusion injury than ischemia injury.

Stroke is an extremely variable clinical condition; the time until the restoration of blood flow by thrombolysis and subsequent delivery of oxygen and nutrients to the ischemic brain varies. Therefore, it is vital that potential therapeutic compounds are able to ameliorate the consequences of permanent occlusion in an animal model [24]. In contrast to transient ischemia, the infarct area was more apparent after permanent MCAO. However, STVNA at a dose of 10 mg/kg, the most effective dose in transient ischemia, still produced a modest effect in the permanent occlusion model (46.3% reduction in infarct size in transient MCAO versus 40% reduction in permanent MCAO). These results indicate that STVNA is active in a model of ischemia without reperfusion and that this effect is not dependent on reperfusion time, which reinforces the interest in STVNA for stroke.

The mechanisms of the protective effects of STVNA have been recently investigated. Some studies had previously reported that STV protects against heart ischemia [25] and cerebral ischemia [14]. It has also been demonstrated that pretreatment with isosteviol enhances the expression of the antiapoptosis factor Bcl-2 and inhibits the expression of NF-kB and COX-2. It also increases SOD and GSH-PX activity and decreases MDA content of the myocardium induced by ischemia-reperfusion in anesthetized rats. It had examined the anti-inflammatory activity of isosteviol using the mouse ear inflammatory test induced by 12-O-tetradecanoylphorbol-13-acetate. The pretreatment with isosteviol resulted in a marked reduction in ear disk edema, with an inhibitory effect of 53% [12]. Oxidation stress and the inflammatory response play very important roles in the cascade after cerebral ischemia, and they can also induce the apoptosis of neurons. As shown in Figure 2, our study demonstrated that edema was inhibited in the ischemia/reperfusion injury acute response period. Apoptosis of neurons was also reduced after STVNA treatment (Figure 6(d)). Therefore, STVNA is a potential neuroprotectant in in vivo models of cerebral ischemia and can act on multiple pathways in the ischemic cascade, suggesting that STVNA might have a greater chance of success in interrupting ischemic injury in acute stroke.

Although many compounds with neuroprotective action are in various stages of the drug discovery, STVNA shows individual advantages. Stevioside (the origin of STVNA) has been extensively used as a sweetener and demonstrated its safety for human [26]. Secondly, isosteviol is lipophilic and can easily diffuse across biological membranes and the BBB. Thirdly, STVNA provides substantial neuroprotection through cerebral perfusion-independent effects and in permanent MCAO models of stroke, unlike tirilazad (a perfusion-dependent neuroprotective agent) [27] and anti-intercellular adhesion molecule-1 antibody [28], which have only been proven effective in tMCAO. Moreover, we compared the neuroprotective potency of STVNA with other established antioxidants with neuroprotective abilities. STVNA resulted in better histological and functional improvement than edaravone (Figures 6 and 3(b)), a ROS scavenger approved in Japan for the treatment of cerebral infarction.

In summary, the present study demonstrated that STVNA can reduce the damage induced by focal cerebral ischemia and was still effective even if the treatment was delayed up to 4 hours after recirculation in temporary focal ischemia or up to 4 hours after artery occlusion in permanent ischemia. Meanwhile, the neuroprotective effectiveness of STVNA is better than edaravone. Therefore, STVNA may be a potent alternative treatment for acute ischemic stroke.

## Figures and Tables

**Table 1:** Physiologic variables in the dose-response study.

	Sham	Vehicle	STVNA, mg/kg				Edaravone
			1	5	10	20	
Before Ischemia (10 minutes)							
BR	63.5 ± 4.5	59.6 ± 5.1	63.5 ± 3.2	64.9 ± 1.4	63.3 ± 2.7	63.7 ± 4.0	62.8 ± 4.2
HR	363 ± 14	376.4 ± 7.6	379.4 ± 8.4	375.7 ± 7.8	380.8 ± 10.5	378.4 ± 11.3	374.2 ± 11
SpO_2_	99.3 ± 0.2	99.3 ± 0.2	99.1 ± 0.3	99.1 ± 0.1	99.2 ± 0.2	99.7 ± 0.1	98.1 ± 0.3
*T*	36.2 ± 0.2	36.2 ± 0.3	36.6 ± 0.2	36.0 ± 0.3	36.4 ± 0.3	36.8 ± 0.2	36.4 ± 0.3
After Ischemia (10 minutes)							
BR	62.7 ± 5.3	69.4 ± 2.3	63 ± 5.1	70.5 ± 3.4	65.4 ± 1.6	68.4 ± 1.5	63.5 ± 2.6
HR	369 ± 10	378.9 ± 4.5	385.3 ± 5.6	376.7 ± 10	378.7 ± 7.8	390.2 ± 7.5	375 ± 8.4
SpO_2_	99.3 ± 0.1	99.3 ± 0.1	99.1 ± 0.2	98.9 ± 0.1	98.9 ± 0.2	99.4 ± 0.2	98.8 ± 0.3
*T*	36.4 ± 0.2	36.7 ± 0.1	36.3 ± 0.3	36.9 ± 0.3	36.5 ± 0.2	37.3 ± 0.1	36.2 ± 0.3
Before reperfusion (10 minutes)							
BR	72.5 ± 2.5	70 ± 1.5	69.5 ± 1.0	71.8 ± 1.5	70.2 ± 2.7	71.3 ± 1.6	67.8 ± 2.3
HR	377 ± 10	390 ± 5.8	400 ± 6.9	405 ± 8.5	400.2 ± 6.6	398.2 ± 9.6	402.5 ± 5.6
SpO_2_	99.4 ± 0.1	99.4 ± 0.1	99.5 ± 0.1	99.4 ± 0.1	99.5 ± 0.1	99.0 ± 0.2	99.4 ± 0.1
*T*	36.2 ± 0.2	37.9 ± 0.2	38.2 ± 0.1	37.9 ± 0.3	38.0 ± 0.1	38.1 ± 0.2	38.3 ± 0.1
After reperfusion (10 minutes)							
BR	73.2 ± 3.2	68.6 ± 2.4	72.6 ± 1.4	72.3 ± 1.9	72.1 ± 3.1	70.5 ± 1.5	69.8 ± 3.2
HR	387 ± 9	401.4 ± 8.4	401.7 ± 2.9	397.1 ± 5.5	396.7 ± 7.8	408.7 ± 4.1	394 ± 10
SpO_2_	99.3 ± 0.1	98.3 ± 0.1	98.7 ± 0.4	99.4 ± 0.1	99.5 ± 0.1	99.7 ± 0.1	99.4 ± 0.1
*T*	37.5 ± 0.1	37.6 ± 0.3	37.9 ± 0.2	37.8 ± 0.1	37.6 ± 0.1	37.0 ± 0.1	37.8 ± 0.2

All of the data were shown as the mean ± the SEM (*n* = 8). BR: breathing rate (breaths/min); HR: heart rate (beats/min); *T*: temperature (°C); SpO_2_: Pulse Oximeter Oxygen Saturation (%).
